# The development of the Cross‐Cultural Family Wellbeing scale for Dementia (CCFW‐D)

**DOI:** 10.1002/alz70858_106512

**Published:** 2025-12-26

**Authors:** Najoua Lazaar

**Affiliations:** ^1^ Erasmus Medical Center, Rotterdam, Netherlands

## Abstract

**Background:**

While current clinical trials and intervention studies mainly use indicators of an individual's quality of life (QoL) as outcomes, individuals from collectivistic cultures may prioritize the wellbeing of the family as a collective over their individual wellbeing. The goal of this study was thus to develop an instrument to measure family wellbeing.

**Method:**

Items were drafted using existing scales (e.g. Catalight wellbeing scale) and by generating new items based on clinical experiences in the multicultural memory clinic. In the pilot stage, individuals from diverse cultural backgrounds were asked to provide feedback on the cultural and linguistic appropriateness of the scale and suggest additional items. A caregiver of a person with dementia also provided extensive feedback. The scale was translated to Turkish and Moroccan languages (*Darija* and *Tamazight)*. Subsequently, the wellbeing scale was administered to caregivers with diverse backgrounds in a study including other burden and quality of life indicators and an acculturation and social identity scale.

**Result:**

The feedback from individuals with diverse linguistic/cultural backgrounds showed that certain items may not be cross culturally applicable or commonly used in certain caregiving contexts. For instance, in the Moroccan language the concept of a peer support group is unfamiliar. Similarly, there is no specific word for ‘caregiver’ and instead is described as ‘the one who takes care of the person’. In the same way ‘emotional support’ has no specific term and is described as ‘someone you can share your emotions with’. Caregiver input revealed that the positive wording of the questions may lead caregivers to feel that it is the norm for processes to run smoothly within a family (potentially impacting answer tendencies or increasing burden). They also suggested it was unclear how to take the person with dementia into consideration, particularly when evaluating family communication. Data on the Cross‐Cultural Family Wellbeing scale's association with individual caregiver burden and quality of life will be presented at the conference.

**Conclusion:**

This promising new family wellbeing scale covers an important gap in dementia trial/intervention outcome assessment in diverse populations. It may also help identify families requiring additional support from a systemic perspective.

